# 

**Published:** 1846-02-01

**Authors:** 


☞ Notices of our exchange Journals in continuation of the article in our 7th number are deferred for want of space. The following publications are acknowledged: Monthly Miscellany, and Journal of Health, Boston, Mass; Reciprocal obligations of Professor and Pupils, an introductory lecture by T. D. Mitchell, Prof. of materia medica in Transylvania University; Introductory lecture by Prof. Bedford, New-York University Med. dept.; Medical and Surgical Reporter; New-York Journal of Medicine and collateral Sciences; Illinois Med. and Surg. Journal; Circular and Catalogue of Castleton, Vt. Med. School; do. of Geneva, N. Y.; Bulletin of Med. Science; Western Lancet; President Pierce's address to the officers and students of the Cleveland Med. College.

